# Difficult Delivery and Neonatal Resuscitation: A Novel Simulation for Emergency Medicine Residents

**DOI:** 10.5811/westjem.2019.10.43913

**Published:** 2019-12-09

**Authors:** Jillian Elizabeth Nickerson, Taryn Webb, Lorraine Boehm, Hayley Neher, Lillian Wong, Julia LaMonica, Suzanne Bentley

**Affiliations:** * Children’s National Medical Center, Department of Emergency Medicine and Trauma Services, Washington, District of Colombia; † Icahn School of Medicine at Mount Sinai, Department of Emergency Medicine, New York, New York; ‡ Elmhurst Hospital Center, Simulation Center, Elmhurst, New York; § Elmhurst Hospital Center, Department of Emergency Medicine, Elmhurst, New York

## Abstract

**Introduction:**

Newborn delivery and resuscitation are rare, but essential, emergency medicine (EM) skills. We evaluated the effect of simulation on EM residents’ knowledge, confidence, and clinical skills in managing shoulder dystocia and neonatal resuscitation.

**Methods:**

We developed a novel simulation that integrates a shoulder dystocia with neonatal resuscitation and studied a convenience sample of EM residents. Each 15-minute simulation was run with one learner, a simulated nurse, and a standardized patient in situ in the emergency department. The learner was required to reduce a shoulder dystocia and then perform neonatal resuscitation. We debriefed with plus/delta format, standardized teaching points, and individualized feedback. We assessed knowledge with a nine-question multiple choice test, confidence with five-point Likert scales, and clinical performance using a checklist of critical actions. Residents repeated all measures one year after the simulation.

**Results:**

A total of 23 residents completed all measures. At one-year post-intervention, residents scored 15% higher on the knowledge test. All residents increased confidence in managing shoulder dystocia on a five-point Likert scale (1.4 vs 2.8) and 80% increased confidence in performing neonatal resuscitation (1.8 vs 3.0). Mean scores on the checklist of critical actions improved by 19% for shoulder dystocia and by 27% for neonatal resuscitation.

**Conclusion:**

Implementing simulation may improve EM residents’ knowledge, confidence, and clinical skills in managing shoulder dystocia and neonatal resuscitation.

## BACKGROUND

Newborn deliveries complicated by shoulder dystocia and the need for neonatal resuscitation occur rarely in the emergency department (ED), but managing these cases are essential skills for emergency physicians. Shoulder dystocia occurs as infrequently as 0.2% of vaginal deliveries in obstetrical literature.^1^ Given that a small percentage of total deliveries occur in the ED, it is uncommon for emergency medicine (EM) residents to manage shoulder dystocia in the ED during their training.^2^ About 10% of neonates require support, and about 1% require resuscitation.^3^ ED deliveries have a higher associated morbidity and may be more likely to require resuscitation; however, performing neonatal resuscitation in the ED is a rare event for individual providers.^4^

The Accreditation Council for Graduate Medical Education (ACGME) requires EM residents to perform 10 low-risk, normal spontaneous vaginal deliveries to graduate. There are no formal teaching requirements, however, for difficult deliveries, such as deliveries complicated by shoulder dystocia.^5^ A recent needs assessment of EM residents demonstrated a lack of knowledge and comfort in obstetrical emergencies, indicating a need for increased education in this area.^6^ A survey of EM program directors (PD) supported these findings, identifying a lack of formal education in obstetrics and a concern from PDs about their graduating residents’ level of preparedness for obstetrical emergencies, specifically for shoulder dystocia.^7^ In a needs assessment of our own residency, we found that 75% of graduating residents lacked confidence in their ability to manage difficult deliveries.

There is no ACGME educational requirement for EM residents to learn neonatal resuscitation.^5^ Although residents become certified in Advanced Cardiac Life Support and Pediatric Advanced Life Support, most do not take the Neonatal Resuscitation Program (NRP), the equivalent course for neonatal resuscitation. A recent trial showed that EM residents lack confidence in leading neonatal resuscitations.^8^ A needs assessment of our residency found that all graduating residents lacked confidence in leading neonatal resuscitations.

Simulation can help fill in training deficits where clinical exposure is rare. Obstetrics and gynecology research has demonstrated the utility of simulation to teach and maintain knowledge, confidence, and clinical skills for difficult deliveries.^9^,^10^ Pediatrics literature also shows an improvement in confidence after simulation of neonatal resuscitation.^11^ A recent randomized control trial of EM residents demonstrated that a simulation curriculum could improve clinical performance of neonatal resuscitation.^8^ Another study showed that simulation training could improve EM faculty knowledge of neonatal resuscitation.^12^

Only one published study has combined shoulder dystocia and neonatal resuscitation in the same simulation case. That study evaluated the feasibility and clinical accuracy of a simulation case designed for medical students that combined a shoulder dystocia with neonatal resuscitation.^13^ We are not aware of any studies that combine delivery complicated by shoulder dystocia with an infant born requiring neonatal resuscitation in a simulation for EM residents despite the need for emergency physicians to integrate these two skills in real patient encounters.

The Society for Academic Emergency Medicine (SAEM) Technology in Medical Education Committee consensus group recommended precipitous and difficult vaginal deliveries, as well as newborn resuscitation, as high-priority areas of EM training.^14^

## OBJECTIVES

This study seeks to evaluate whether an in situ simulation can improve EM residents’ knowledge, confidence, and clinical skills in performing maneuvers to reduce a shoulder dystocia and then leading a neonatal resuscitation.

## CURRICULAR DESIGN

There is no standard education that teaches EM residents how to manage difficult deliveries, such as deliveries complicated by a shoulder dystocia, or to lead a neonatal resuscitation. We conducted a needs assessment of eight graduating postgraduate year (PGY) 4 residents’ confidence with these skills, and found that the majority (75%) noted feeling “not confident at all” or “barely confident” in reducing a shoulder dystocia and no residents felt “confident” or “very confident” in leading a neonatal resuscitation. From these data, we designed an intervention to address this curricular need in our program.

We developed a novel simulation session integrating a newborn delivery complicated by a shoulder dystocia with a subsequent need for neonatal resuscitation. The 15-minute simulation was run with one learner, an embedded simulation nurse, and a standardized patient in the ED setting. The “patient” was a live standardized patient actor using a PROMPT flex birthing simulator (Laerdal Medical, Stavenger, Norway) and a Code Blue Newborn (Gaumard Scientific, Miami, FL). A convenience sample of residents across all years (PGY 1–4) consented to participate and were sampled while working clinically in the ED. The learner was required to perform critical actions to reduce a shoulder dystocia to deliver an apneic neonate and then perform neonatal resuscitation per NRP guidelines (Table 1).

The simulation case was developed from prior cases in collaboration with content and simulation experts from EM, obstetrics, and neonatology. The integrated case was piloted on six participants including a resident from each PGY year, a senior EP assistant, and an attending EP. The case was adapted based on feedback from participants and simulation experts prior to study initiation.

Following completion of the simulated case, the learners were debriefed using the PEARLS Healthcare Debriefing Tool with plus/delta format by trained simulation leaders.^15^ Learners reviewed standardized teaching points that emphasized key maneuvers to reduce a shoulder dystocia and critical steps to performing neonatal resuscitation from NRP. Additionally, learners received individualized feedback based on their specific questions and performance.

Residents were surveyed on knowledge and confidence before participating in the simulation and one year after they completed the simulation. We also questioned residents about the number of deliveries complicated by shoulder dystocia and the number of neonatal resuscitations they had participated in. We assessed knowledge using a nine-question multiple choice test adapted from tools used at our simulation center to evaluate knowledge from a course on shoulder dystocia for obstetrical providers and a course about neonatal resuscitation for pediatric providers. Our experts selected the questions that were most pertinent to the EM provider caring for these conditions. We assessed confidence using five-point Likert scales. We also surveyed residents about their experience participating in the simulation.

Clinical performance was scored using a checklist of critical actions. A team comprised of a fellowship-trained simulation expert, an EM attending, an obstetrical attending, and a neonatal intensivist reviewed the critical actions from our institution’s shoulder dystocia management course, NRP guidelines,^16^ and the checklists used in a published, integrated simulation for medical students.^13^ From these tools, we developed our own checklist of critical actions (shown in Table 1) using an iterative process and focusing on the skills important for the EM provider. We used expert judgment to ensure content validity. Those rating clinical performance were trained via frame-of-reference training.^17^ Sample cases were scored and compared until an acceptable inter-rater reliability was reached. All study cases were scored by two independent observers with a strong inter-rater reliability (kappa 0.84).

The simulation was repeated one year after the initial simulation with a convenience sample of two classes of residents to evaluate retention and whether the simulation impacted clinical skills. This study was approved by the Icahn School of Medicine at Mount Sinai institutional review board.

## IMPACT/EFFECTIVENESS

### Demographics

A total of 52 residents completed the simulation, spread across four classes of residents: PGY-1, 25% (13); PGY-2, 29% (15); PGY-3, 25% (13); and PGY-4, 21% (11). We repeated the simulation one year later with residents who were PGY-2 or PGY-3 during the initial simulation. Of the 27 eligible residents, 23 residents (9 PGY-3 and 14 PGY-4) completed the repeat simulation.

### Baseline

At baseline, interns (n = 13) demonstrated a knowledge deficit compared to PGY 2–4 (n = 39) classes (53% vs 66%). We did not find a difference in scores between the senior residents. On average, prior to any teaching, residents (n = 53) scored 69% (12.5/18) for shoulder dystocia and 63% (9.5/15) for neonatal resuscitation on the checklist of critical actions. Although our numbers were small, we could not discern a difference in performance between junior and senior residents.

### Perception

Overall, residents (n = 53) reported positive views of the simulation. The majority (93%) said the overall learning value of the case was “excellent” or “very good.” Of the 53 residents who completed the evaluation of the simulation, 17 (32%) provided a qualitative comment. Of those, 76% (13) specifically remarked that simulation of shoulder dystocia and/or neonatal resuscitation was useful for their training. This sentiment is exemplified by one participant’s comment: “This topic is incredibly scary and is something we barely have real experience with. The ability to do this scenario in a safe and controlled setting was delightful.”

### Knowledge

One year after completing the initial simulation and debriefing, residents (n = 23) demonstrated an increase in knowledge scores by 15% (57% pre-simulation vs 72% post-simulation). The majority (90%) scored at least one point higher on the repeat exam one year after training.

### Confidence

All 23 residents reported improved confidence in managing shoulder dystocia on a five-point Likert scale with one representing no confidence and five representing extreme confidence (mean 1.4 pre-simulation vs 2.8 post-simulation). The majority (80%) reported increased confidence in performing neonatal resuscitation (mean 1.8 vs 3.0) one year after completion of the simulation.

### Clinical Performance

**Figure 1:**
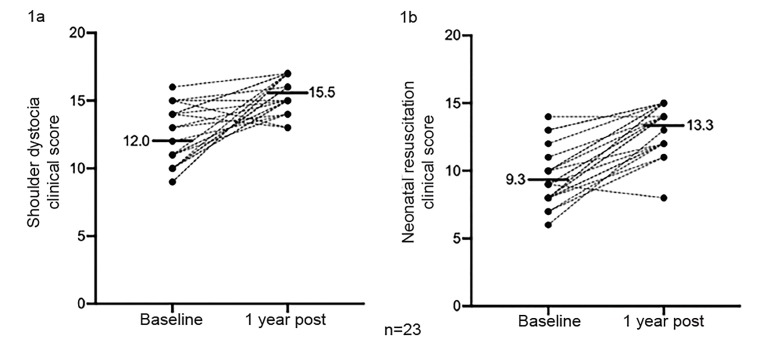
Changes in residents’ clinical scores one year after simulation.

Residents who completed the training (n = 23) had improvements in clinical performance. Shoulder dystocia critical action scores improved from 67% (12.0/18) at baseline to 86% (15.4/18). Similarly, neonatal resuscitation scores improved from 62% (9.3/15) at baseline to 89% (13.3/15) (Figure 1).

Figure 1 shows an increase in clinical scores for both shoulder dystocia (1a) and neonatal resuscitation (1b) from baseline to one year after completing the simulation for the 23 residents who completed both simulations.

### Clinical Exposure

At baseline, 30% (7/23) of residents who completed both pre- and post-simulations reported that they had participated in a delivery complicated by shoulder dystocia, and 52% (12/23) reported that they had participated in a neonatal resuscitation in either a real or simulated case. On reassessment, one year after participating in the simulation of a delivery complicated by shoulder dystocia and an infant requiring neonatal resuscitation, 60% (14/23) of residents indicated that they had participated in the care of a real or simulated patient with shoulder dystocia, and 91% (21/23) indicated that they had participated in a real or simulated neonatal resuscitation. However, no participants reported participating in more than three instances of either pre- or post-intervention.

## DISCUSSION

Previous literature has supported the use of simulation to train obstetrical residents to manage patients with shoulder dystocia^5^,^6^ and EM providers to manage neonatal resuscitation.^4^ Our study builds on previous literature by combining the two skills into one simulation. We also conducted the simulation in the ED setting providing a high level of fidelity to the training for EM providers.

## LIMITATIONS

The small sample size limits our ability to statistically analyze our data. We evaluated residents over time; therefore, their performance could have been impacted by exposure to patients in the ED with these conditions, outside reading, and lectures in addition to our intervention. While we adapted our tools from previously used tools, these have not been formally assessed for validity. Two independent observers graded each participant with the checklist of critical actions; however, there remains some subjectivity to the scores.

## CONCLUSION

EM residents lack confidence and demonstrate knowledge deficits in managing shoulder dystocia and performing neonatal resuscitation. Implementing simulation may improve knowledge, confidence, and clinical performance in managing shoulder dystocia and performing neonatal resuscitation. By implementing simulations that combine difficult deliveries with neonatal resuscitation, a new minimum standard for education in these areas for EM residents can be established.

## Figures and Tables

**Table 1:** Shoulder dystocia/neonatal resuscitation performance observation tool.

Shoulder dystocia
Identifies precipitous labor Poor: delay in examining, incomplete setup Average: examines and identifies crowning head with some delay, some hesitation in determining delivery necessity or with what supplies are necessary Excellent: quickly examines, correctly identifies head crowning, and calls for team and necessary supplies to deliver
Checks for cord Poor: requires prompting from nurse to evaluate for nuchal cord Average: some delay in assessing for nuchal cord Excellent: sweeps for cord, finds nuchal cord, reduces successfully
Identifies shoulder dystocia Poor: requires prompting from nurse or patient to identify shoulder dystocia Average: some delay in identifying shoulder dystocia, fails to note time Excellent: quickly determines and states aloud that patient has a shoulder dystocia, asks nurse to record time, tells mom to stop pushing
Calls for help Poor: failure to call services Average: some delay in calling for help, or calling for only one service Excellent: quickly calls for obstetrics and pediatrics for help
Initiates McRoberts maneuver Poor: Cannot perform suprapubic pressure even with prompting Average: can direct team to perform McRoberts but does not recall name or some difficulty with procedure Excellent: smoothly calls for McRoberts maneuver and directs team to perform appropriately
Initiates suprapubic pressure Poor: Cannot perform suprapubic pressure even with prompting Average: calls for suprapubic pressure but some delay or some difficulty with procedure or does not know directionality Excellent: smoothly and quickly calls for suprapubic pressure and can describe to team how to perform appropriately

Neonatal resuscitation

Dries and stimulates newborn appropriately Poor: fails to dry and stimulate Average: some delay or slightly clumsy, requires nudge Excellent: calls for and smoothly and quickly dries, removes wet blankets, and stimulates newborn
Adequately evaluates respirations, heart rate, and color Poor: does not complete without prompting Average: calls out need for evaluation, some delay in calculating, uses umbilical cord for heart rate Excellent: quickly calls out need for evaluation of heart rate, respirations and notes color
Identifies need for and initiates respirations correctly Poor: does not identify need for positive pressure ventilation without prompting or fails to achieve proper seal or evaluate for chest size Average: some delay, or mild deficiencies or inconsistency Excellent: quickly calls out for positive pressure ventilation, selects correct mask, correctly seals mask and bags 1 breath every 3 seconds with evaluation for chest rise
Correctly identifies need for intubation and intubates successfully Poor: unable to identify need for intubation, necessary materials, or successfully intubate Average: slight delay or some difficulty with calling for sizes of materials but ultimately successful intubation Excellent: identifies need for intubation in a timely manner, calls for correct size of blade and endotracheal tube, intubates successfully with tube at appropriate depth, evaluates for bilateral breath sounds and chest rise
Identifies need for and initiates compressions correctly when heart rate remains <60 Poor: does not identify need for compressions without prompting or poor quality Average: some delay, mild inconsistency or deficiency in positioning, rate or depth Excellent: calls out for chest compressions, delivers compressions at correct rate and depth

Checklist of critical actions and scoring guide used to evaluate residents’ ability to reduce a shoulder dystocia and perform a neonatal resuscitation.
